# EGFR transactivation contributes to neuroinflammation in *Streptococcus suis* meningitis

**DOI:** 10.1186/s12974-016-0734-0

**Published:** 2016-10-19

**Authors:** Xiao-Pei Yang, Ji-Yang Fu, Rui-Cheng Yang, Wen-Tong Liu, Tao Zhang, Bo Yang, Ling Miao, Bei-Bei Dou, Chen Tan, Huan-Chun Chen, Xiang-Ru Wang

**Affiliations:** 1 State Key Laboratory of Agricultural Microbiology, College of Veterinary Medicine, Huazhong Agricultural University, Wuhan, Hubei 430070 China; 2 The Cooperative Innovation Center for Sustainable Pig Production, Huazhong Agricultural University, Wuhan, Hubei 430070 China; 3 Key Laboratory of Development of Veterinary Diagnostic Products of Ministry of Agriculture, Huazhong Agricultural University, Wuhan, Hubei 430070 China

**Keywords:** *Streptococcus suis*, Brain microvascular endothelial cell, EGFR, Neuroinflammation

## Abstract

**Background:**

*Streptococcus suis* serotype 2 (SS2) is an important zoonotic bacterial pathogen in both humans and animals, which can cause high morbidity and mortality. Meningitis is one of the major clinical manifestations of SS2 infection. However, the specific process of SS2 meningitis and its molecular mechanisms remain unclear. Epidermal growth factor receptor (EGFR) has been reported to initiate transduction of intracellular signals and regulate host inflammatory responses. Whether and how EGFR contributes to the development of *S. suis* meningitis are currently unknown.

**Methods:**

The tyrosine phosphorylation of cellular proteins, the transactivation of EGFR, as well as its dimerization, and the associated signal transduction pathways were investigated by immunoprecipitation and western blotting. Real-time quantitative PCR was used to investigate the transcriptional level of the ErbB family members, EGFR-related ligands, cytokines, and chemokines. The secretion of cytokines and chemokines in the serum and brain were detected by Q-Plex™ Chemiluminescent ELISA.

**Results:**

We found an important role of EGFR in SS2 strain SC19-induced meningitis. SC19 increasingly adhered to human brain microvascular endothelial cells (hBMEC) and caused inflammatory lesions in the brain tissues, with significant induction and secretion of proinflammatory cytokines and chemokines in the serum and brains. SC19 infection of hBMEC induced tyrosine phosphorylation of cellular EGFR in a ligand-dependent manner involving the EGF-like ligand HB-EGF, amphiregulin (AREG), and epiregulin (EREG) and led to heterodimerization of EGFR/ErbB3. The EGFR transactivation did not participate in SS2 strain SC19 adhesion of hBMEC, as well as in bacterial colonization in vivo. However, its transactivation contributed to the bacterial-induced neuroinflammation, via triggering the MAPK-ERK1/2 and NF-κB signaling pathways in hBMEC that promote the production of proinflammatory cytokines and chemokines.

**Conclusions:**

We investigated for the first time the tyrosine phosphorylation of cellular proteins in response to SS2 strain SC19 infection of hBMEC and demonstrated the contribution of EGFR to SS2-induced neuroinflammation. These observations propose a novel mechanism involving EGFR in SS2-mediated inflammatory responses in the brain, and therefore, EGFR might be an important host target for further investigation and prevention of neuroinflammation caused by SS2 strains.

## Background

*Streptococcus suis* is one of the most prevalent pathogens in swine herds; it can cause a wide variety of life-threatening infections or syndromes in pigs, including septicemia, meningitis, endocarditis, arthritis, and even sudden death, resulting in serious economic losses in the pig industry. It is also an emerging zoonotic pathogen with the ability to induce meningitis, endocarditis, and streptococcal toxic shock-like syndrome (STSLS) in humans, which usually result from direct contact with infected pigs or pig products [1]. So far, *S. suis* infection in humans has been largely reported in Asian countries, as well as in North and South America, Australia, New Zealand, and several European countries [2–4]. Among the 33 *S. suis* serotypes, *S. suis* 2 (SS2) is the most prevalent and virulent in pigs and humans [5]. The outbreak of SS2 in 2005 in China resulted in more than 200 cases of human infection, with a fatality rate reaching 20 % [2]. Meningitis is an important clinical manifestation of SS2 infection, in which more than 50 % of patients suffer from hearing loss sequelae [1, 5]. Recently, SS2 has emerged as the most frequent pathogen responsible for bacterial meningitis in adults in southern Vietnam, the second-most prevalent in Thailand, as well as the third-most-common cause of community-acquired bacterial meningitis in Hong Kong [3, 4, 6–8]. However, little is known about how SS2 strains penetrate the blood-brain barrier (BBB) and cause meningitis.

Innate immunity is an essential host defense against infection by pathogenic microorganisms, in which cytokines function as an indispensable component of the defense. Previous literature has demonstrated that SS2 infection in mice could induce the strong generation of diverse proinflammatory cytokines and chemokines in blood, including tumor necrosis factor alpha (TNF-α), interleukin (IL)-6, IL-12, interferon (IFN)-γ, monocyte chemoattractant protein (MCP)-1, chemokine (C-X-C motif) ligand 1 (GRO-α/CXCL1), C-C motif chemokine ligand 5 (CCL5/RANTES) [9].The excessive production of proinflammatory cytokines was considered to be the most important cause of SS2 meningitis and septicemia, as well as STSLS [9, 10]. In recent years, additional SS2 components have been reported to mediate the release of proinflammatory cytokines and contribute to the development of meningitis, including capsular polysaccharide (CPS), suilysin, muramidase-released protein (MRP), and SspA [4, 5, 11, 12]. However, the specific host molecules participating in SS2 meningitis, as well as regulating the generation of proinflammatory cytokines, were poorly understood.

Epidermal growth factor receptor (EGFR) is recognized as an important initiator manipulated by diverse pathogens for their survival in the host and inducing inflammatory responses [13, 14]. EGFR belongs to the ErbB family of receptor tyrosine kinases, which consists of four closely related members (ErbB1/EGFR, ErbB2, ErbB3, ErbB4) [15–17]. It is initially expressed in the plasma membrane in an inactive form and transactivated through certain kinases and/or after binding to its specific ligands, which are produced as transmembrane precursors and released after proteolytic cleavage [15–18]. EGFR transactivation leads to either homodimerization or heterodimerization that stimulates the intrinsic tyrosine kinase activity and triggers autophosphorylation of specific tyrosine residues within the cytoplasmic domains, therefore promoting the activation of its downstream signaling cascades [15, 19]. Although EGFR has been largely reported in the field of cancer because of its involvement in cell proliferation, migration, and invasion [20, 21], an increasing number of studies have supported its diverse roles in pathogenic bacterial infections, including *Haemophilus influenza*, *Klebsiella pneumonia*, *Neisseria gonorrhoeae*, *Pseudomonas aeruginosa*, and *Helicobacter pylori* infections [14, 22–24]. However, the specific role of EGFR in SS2 infection, especially its association with SS2-induced meningitis, is completely unclear.

As the most distinct and indispensable structural and functional component of the BBB, brain microvascular endothelial cells (BMEC) prevent circulating pathogens from entering the brain, thus maintaining central nervous system (CNS) homeostasis [25]. To further determine the mechanism of SS2-induced meningitis, we sought to identify and characterize the potential host targets that participate in SS2 infection of BMEC, as well as induction of proinflammatory cytokines and chemokines. We provide evidence that EGFR acts as an essential host target in SS2 interaction with the BBB and an important molecular switch that additionally controls the production of proinflammatory cytokines. These findings provide evidences supporting the role of EGFR in SS2-mediated neuroinflammation, which will expand our knowledge on SS2-induced CNS dysfunction.

## Methods

### Bacterial strains and cell culture

SS2 strain SC19, originally isolated from a pig brain during the *S. suis* outbreak in Sichuan Province in China in 2005 [26], was cultured in TSB broth or on TSA plates (Difco Laboratories, Detroit, MI, USA) with 10 % newborn bovine serum at 37 °C with appropriate antibiotics unless otherwise specified.

The human BMEC cell line (hBMEC) was kindly provided by Prof. Kwang Sik Kim in Johns Hopkins University School of Medicine [27, 28] and cultured in RPMI 1640 medium with 10 % heat-inactivated fetal bovine serum (FBS), 10 % Nu-serum, 2 mM L-glutamine, 1 mM sodium pyruvate, nonessential amino acids, vitamins, and penicillin and streptomycin (100 U/mL) in a 37 °C incubator under 5 % CO_2_ [27]. Confluent cells were washed three times with Hanks’ Balanced Salt Solution (Corning Cellgro, Manassas, VA, USA) and starved in serum-free medium for 16–18 h before further treatment. In some experiments, cells were pretreated with specific inhibitors for the indicated times before challenge.

### Reagents, antibodies, and shRNA plasmids

The EGFR tyrosine kinase inhibitor AG1478, nuclear factor kappa B (NF-κB) inhibitors CAY10657 and BAY-11072, and ERK1/2 inhibitor U0126 were purchased from Cayman Chemical Company (Ann Arbor, MI, USA). Protein A + G agarose beads and MTT Cell Proliferation Assay Kit were obtained from Beyotime (Shanghai, China). Horseradish peroxidase (HRP)-conjugated anti-phosphotyrosine (4G10) antibody was purchased from EMD Millipore Corporation (Temecula, CA, USA). Anti-phosphotyrosine antibody was purchased from Abcam (Cambridge, MA, USA). Anti-NF-κB p65, anti-phospho-p65, anti-IκBα, anti-ErbB2, anti-ErbB3, HRP-conjugated anti-rabbit IgG, and HRP-conjugated anti-mouse IgG antibodies were all purchased from Cell Signaling Technology (Danvers, MA, USA). Anti-ErbB4 antibody was purchased from Proteintech (Chicago, IL, USA). Anti-β-actin antibody was purchased from HuaAn Biotechnology Co., Ltd. (Hangzhou, China). The Lipofectamine 3000 transfection reagent was obtained from Invitrogen (Carlsbad, CA, USA). Puromycin was purchased from Corning Cellgro. The ErbB3 shRNA plasmid and control shRNA plasmid-A were purchased from Santa Cruz Biotechnology (Dallas, TX, USA).

### Bacterial adhesion assays in hBMEC

The determination of adhesion of SS2 strain SC19 to hBMEC was performed as previously described [29, 30]. Briefly, SC19 strain was grown overnight, centrifuged, and resuspended in experimental medium (M199-Ham F12 [1:1] medium containing 5 % heat-inactivated FBS) at 10^7^ CFU/ml. A confluent monolayer of hBMEC grown in a 24-well plate was infected with bacteria at a MOI of 10 for the indicated time followed by extensive washing to remove the unbound bacteria. Cells were lysed with 0.025 % Triton X-100 for 10 min. Counts of adherent bacteria were determined by plating at appropriate dilutions. The assay was performed independently in triplicate.

### Immunoprecipitation and western blotting

The hBMEC were seeded at 1 × 10^6^ cells/100 mm^2^ dish and cultured until confluent. Serum-starved cells were stimulated with SC19 at a MOI of 10 for the indicated times, and cell lysates were then collected for immunoprecipitation and western blotting analysis as previously described [19, 31]. The densimetric analysis was performed using ImageJ software.

### Transfection

The hBMEC were seeded in 6-well plates and grown to 70 % confluence and transfected with either the shRNA targeting ErbB3 or the control shRNA plasmid-A using the Lipofectamine 3000 transfection reagent according to manufacturers’ instructions. Briefly, liquid A (containing 5 μg plasmid, 10 μL P3000, and 250 μL opti-MEM) and liquid B (containing 7.5 μL Lipo3000 and 250 μL opti-MEM) were gently mixed and incubated at room temperature for 5 min. This suspension was then added dropwise to the cells and incubated at 37 °C with 5 % CO2 for 4–6 h. Fresh medium containing puromycin (100 μg/ml) was then applied to screen and maintain the positively transfected cells for 2–3 weeks.

### MTT assay

The hBMEC was seeded in 96-well plates at 5 × 10^3^ cells per well in 100 μL medium and incubated for 24 h; 5 μM AG1478 was added to the wells and incubated for various times as indicated. The supernatant of each well was removed, and MTT dissolved in serum-free medium was added and incubated for a further 4 h. To each well, 100 μL of MTT solvent was then added, and the plate was wrapped in foil and shaken for 15 min. Finally, the absorbance at 570 nm of each well was determined.

### Immunofluorescence microscopy

The hBMEC was seeded at 1 × 10^5^ cells onto coverslips in 24-well plates and cultured for 24 h. Cells were challenged with SS2 at a MOI of 10 for 3 h, washed and fixed with 4 % paraformaldehyde. The fixed cells were subsequently incubated with primary anti-p65 antibody and then with FITC-labeled goat anti-mouse IgG antibody. The plate was mounted and visualized using fluorescence microscopy.

### Animal infection

Five-week-old female CD1 mice obtained from the Center for Disease Control (Hubei Province, China) were used for our animal infection assays. Mice were challenged with the SC19 strain at 2 × 10^8^ CFUs in sterile PBS through the tail vein. At the indicated time points, mice were anesthetized and peripheral blood harvested for serum collection. Subsequently, mice were perfused with heparin solution (10 U/ml) in PBS as previously described [32] and processed for further assays.

### Histopathological examination

Brain samples were collected at the indicated time points post infection and fixed in 4 % paraformaldehyde solution for over 24 h. The fixed-tissues were paraffin embedded for H&E staining following standard procedures [33].

### Reverse transcription and real-time PCR

TRIzol reagent (Invitrogen) was used to isolate total RNA from the infected hBMEC; 1 μg of total RNA was used for cDNA synthesis using the PrimeScript™ RT reagent kit with gDNA Eraser (Takara, Shiga, Japan). Contaminating DNA was removed by the gDNA Eraser treatment during the reverse transcription. Quantitative PCR was conducted with ViiA™ 7 Real-Time PCR System (Applied BioSystems, Foster City, CA, USA) using Power SYBR Green PCR master mix (Applied BioSystems) according to the manufacturers’ instructions. Primers for the real-time PCR are listed in Table 1.

**Table 1:** Primers used in this study

Gene	Forward (5′–3′)	Reverse (5′–3′)	Species
IL-6	GGACTGATGCTGGTGACAAC	GGAGTGGTATCCTCTGTGAAGT	Murine
IL-1α	CTGAAGAAGAGACGGCTGAGTT	CTGGTAGGTGTAAGGTGCTGAT	Murine
MCP-1	ACTCACCTGCTGCTACTCAT	TGTCTGGACCCATTCCTTCTT	Murine
MIP-2	TGACTTCAAGAACATCCAGAG	CCTTGCCTTTGTTCAGTATCT	Murine
GRO- α	TGGCTGGGATTCACCTCAA	GTGGCTATGACTTCGGTTTGG	Murine
β-actin	GTCCCTCCTCTGATACCTTCCTC	CTGGCAGTGTCATTCACATCTTTCT	Murine
EGFR	TACAGACCCAAGAGCAGCA	AGCCGTACATAGATCCAGAA	Human
ErbB2	GTCCCTCCTCTGATACCTTCCTC	CTGGCAGTGTCATTCACATCTTTCT	Human
ErbB3	TACAGACCCAAGAGCAGCA	AGCCGTACATAGATCCAGAA	Human
ErbB4	GTCCCTCCTCTGATACCTTCCTC	CTGGCAGTGTCATTCACATCTTTCT	Human
MIP-2	AGTGTGAAGGTGAAGTCC	CTTTCTGCCCATTCTTGAG	Human
GRO-α	TGCTGCTCCTGCTCCTGGTA	TGTGGCTATGACTTCGGTTTGG	Human
IL-6	CCTTCGGTCCAGTTGCCTTCT	GAGGTGAGTGGCTGTCTGTGT	Human
MCP-1	ATAGCAGCCACCTTCATT	GCTTCTTTGGGACACTTG	Human
TNF-α	AATGGCGTGGAGCTGAGA	TGGCAGAGAGGAGGTTGAC	Human
IL-8	GACATACTCCAAACCTTTCC	ATTCTCAGCCCTCTTCAAA	Human
GAPDH	TGCCTCCTGCACCACCAACT	CGCCTGCTTCACCACCTTC	Human

### Multiplex cytokine and chemokine assays

Mice were challenged with the SC19 strain as described above. In some groups, mice were pretreated with 10 mg/kg AG1478 intraperitoneally 2 h prior to infection. At the indicated time points, mice were euthanized and serum were prepared. Brain tissue samples were lysed in RIPA buffer with protease inhibitor cocktail and centrifuged at 12,000*g* for 10 min to remove tissue debris. The serum and brain extracts were stored at −80 °C and later used for the measurement of 12 preselected cytokines and chemokines using Q-Plex™ Chemiluminescent ELISA (Quansys Bioscience, Logan, Utah, USA) according to the instructions. The preselected cytokines and chemokines were IL-1α, IL-1β, interleukin-6 (IL-6), IL-10, IL-17, TNF-α, IFN-γ, monocyte chemoattractant protein-1 (MCP-1)/CCL2, RANTES/CCL5, MIP-2/CCL8, GROα/CXCL1, and EOTAXIN/CCL11. Multiplex cytokine and chemokine levels were quantitatively analyzed using Bio-Rad Chemi-Doc XRS camera (Bio-Rad, Hercules, CA, USA) and Q-View™ Software (Quansys Bioscience).

### Statistical analysis

Data were expressed as the mean ± SD. The difference between the two groups was analyzed using Student’s *t* test and GraphPad Prism version 6.0 (GraphPad Software Inc., La Jolla, CA, USA). *p < 0.05 was considered statistically significant; **p < 0.01 and ***p < 0.001 indicated extremely significant differences.

## Results

### SS2 strain SC19 infection could induce a strong neuroinflammatory response

**Fig. 1:**
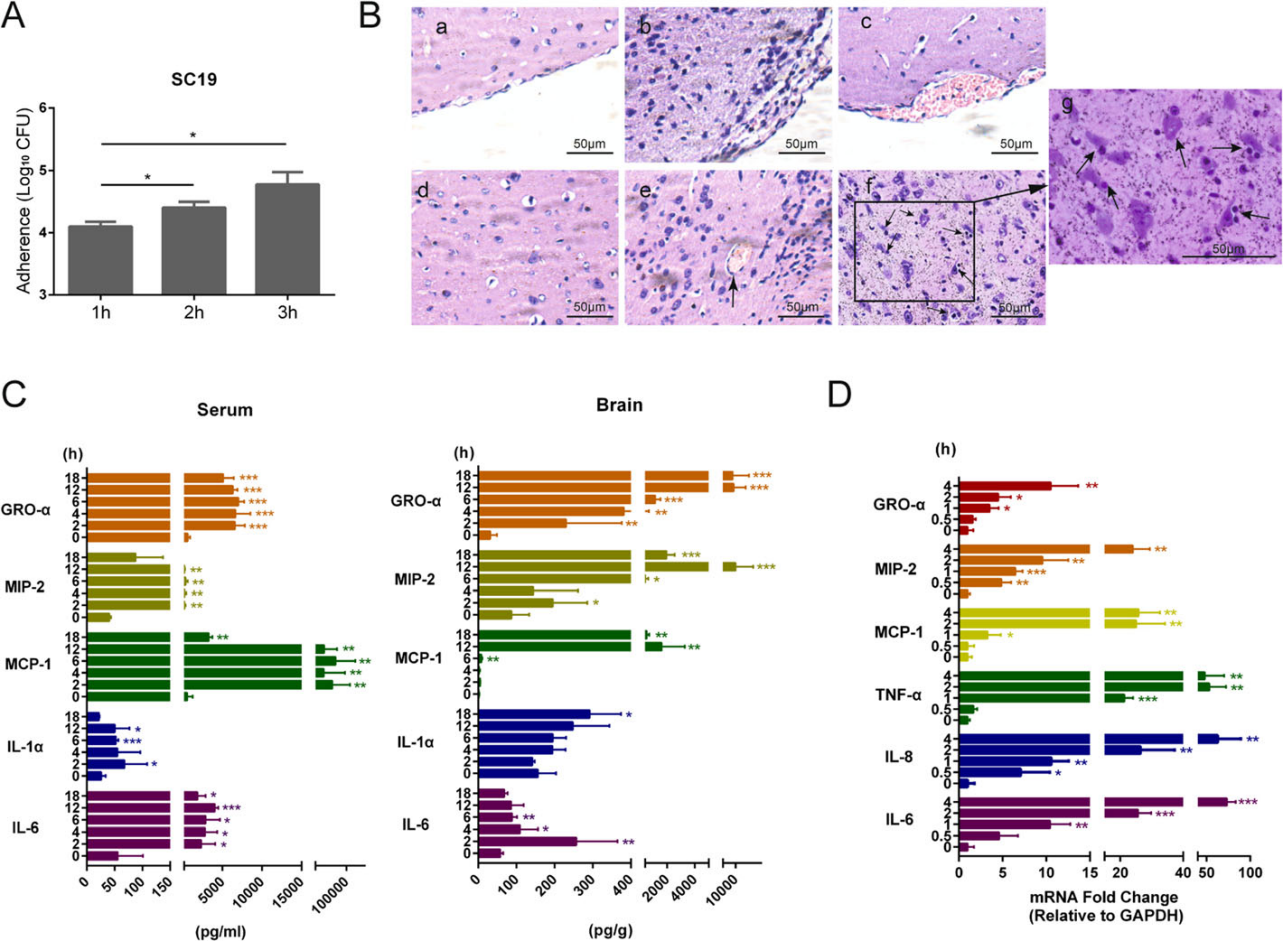
SS2 strain SC19 infection induced a strong neuroinflammation. **a** Increasing adherence of SC19 to hBMEC along with time. **b** 5-week-old CD1 mice were injected intravenously with 2 × 10^8^ CFU SC19 strain. The histopathological changes of the brain in infected mice were investigated. (*a*, *d*) Normal cerebral meninges and brain tissue, (*b*) meningeal thickening, (*c*) subdural hemorrhage, (*e*) perivascular inflammatory infiltrates, (*f*) slight neuronophagia, and (*g*) partial magnification of panel (*f*). *Scale bar* = 50 μm. **c** Brain lysates and serum were harvested at various time points post infection and cytokines production were determined by Q-Plex™ Chemiluminescent ELISA. Results were expressed as the mean ± SD from three infected mice at each time point. Statistical analysis was carried out between the infected group at each time point and the uninfected group (0 h). **d** Total RNAs of infected and uninfected hBMEC were extracted and reversely transcribed. The mRNA levels of cytokines and chemokines were markedly increased in response to SC19 stimulation

We first analyzed SS2 strain SC19 infection of the brain in vitro and in vivo. Using hBMEC in vitro, we observed that SS2 strain SC19 exhibited an increasing adhesion to hBMEC along with infection (Fig. 1a). We did not observe an obvious invasion of SC19 into the cells (data not shown), which is consistent with early reports that *S. suis* strains can adhere to but cannot effectively invade BMEC [34]. In vivo, 5-week-old CD1 mice were challenged with SC19 to induce the occurrence of meningitis, as well as CNS dysfunction. Certain histopathological changes, such as meningorrhagia, meningeal thickening, inflammatory cell accumulation, gliocyte proliferation, and slight neuronophagia (Fig. 1b) were observed in brain tissues in response to SC19 infection, indicating that SS2 strain SC19 infection could lead to histopathological lesions in the brain. In addition, the production of cytokines and chemokines in both brain tissue and serum were determined at the indicated time points. As shown, many proinflammatory cytokines and chemokines (including IL-6, IL-1α, MCP-1, MIP-2, GRO-α) increased rapidly in response to SS2 infection as early as 2 h in the serum and remained elevated until the end of the observation period (Fig. 1c). Other cytokines and chemokines, such as IL-1β, IL-10, IL-17, TNF-α, IFN-γ, RANTES, and EOTAXIN, were also upregulated in the serum upon infection, but to a relatively lesser extent (data not shown). Similarly, these cytokines and chemokines (specifically IL-6, IL-1α, MCP-1, MIP-2, GRO-α) exhibited an increase in the brain. Notably, the chemokines (MCP-1, MIP-2, GRO-α) exhibited a sharp increase almost 6 h later post infection (Fig. 1c). This implies a gradual development of local inflammation based on the increasing systemic inflammatory response. Using real-time PCR, we similarly demonstrated that SS2 strain SC19 infection of hBMEC resulted in a strong production of proinflammatory cytokines and chemokines, including IL-6, IL-8, TNF-α, MCP-1, MIP-2, and GRO-α, in a time-dependent manner (Fig. 1d), suggesting a preliminary inflammatory response at bacterial entry of the BBB.

### SS2 infection of hBMEC induced tyrosine phosphorylation of several cellular proteins, which could be prevented by AG1478

**Fig. 2:**
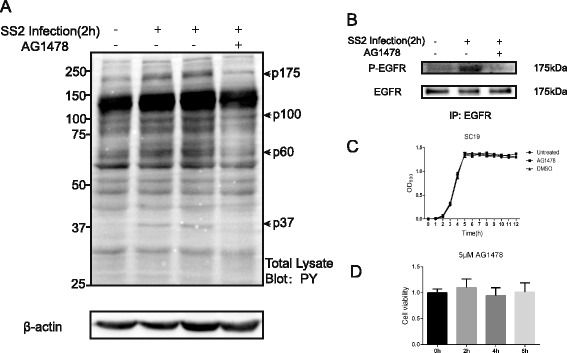
SS2 strain SC19 induces tyrosine phosphorylation of several cellular proteins. **a** Tyrosine phosphorylation of the hBMEC extracts in response to SS2 infection were analyzed by immunoblotting with anti-phosphotyrosine antibody (*PY*). The tyrosine-phosphorylation of several proteins could be markedly inhibited by AG1478. β-actin was analyzed as a normalization control. **b** The hBMEC was lysed and subjected to immunoprecipitation with an anti-EGFR antibody and immunoblotting with the anti-phosphotyrosine mAb 4G10. **c** Bacteria were cultured in TSB containing 10 % newborn bovine serum, and treatment with 5 M AG1478 or vehicle (DMSO) had no influence on bacterial growth at 37 °C. **d** Treatment with 5 μM AG1478 did not influence the hBMEC proliferation, as demonstrated by MTT assay

Tyrosine phosphorylation plays an important role in bacterial pathogen interaction with host cells, and tyrosine phosphorylation of host proteins were reported to be involved in cytoskeletal rearrangement and intracellular signal transduction, as well as the recruitment of the Ser/Thr kinase in several cases [35, 36]. We next focused on intracellular tyrosine phosphorylation to investigate the possible changes of intracellular molecules in response to SS2 infection. Here, serum-starved hBMEC was either infected with SC19 or left uninfected for 2 h, and the tyrosine phosphorylation of intracellular proteins were compared by western blot. As shown in Fig. 2a, SC19 infection of hBMEC induced an obvious tyrosine phosphorylation of several cellular proteins at approximately 180, 100, 60, and 37 kDa, among which the protein at approximately 180 kDa showed the major difference upon SC19 infection. This specific phosphorylation of protein at 180 kDa reminded us of the possibility of the ErbB family members, in which EGFR (ErbB1) was shown to be around 180 kDa and possesses several phosphotyrosine sites. We subsequently pretreated the cells with the EGFR specific inhibitor AG1478 at 5 μM prior to SC19 infection, and we observed that the tyrosine phosphorylation of these identified proteins decreased markedly after AG1478 pretreatment, suggesting the possible involvement of EGFR and its related signaling molecules in SS2 strain infection of hBMEC. To further verify our hypothesis, we investigated EGFR via immunoprecipitation with an anti-EGFR antibody. It is shown in Fig. 2b that SC19 infection induced a prominent phosphorylation of EGFR, while this could be significantly blocked by its inhibitor AG1478. The pretreatment of AG1478 at 5 μM had no effect on the growth of bacteria as assessed by growth curves (Fig. 2c), as well as on cell toxicity as determined by the MTT method (Fig. 2d). Based on these observations, we speculated that EGFR could be one of the major signaling molecules that plays certain roles in SS2 infection of hBMEC.

### SS2 infection of hBMEC results in ligand-dependent activation, as well as the dimerization of EGFR

**Fig. 3:**
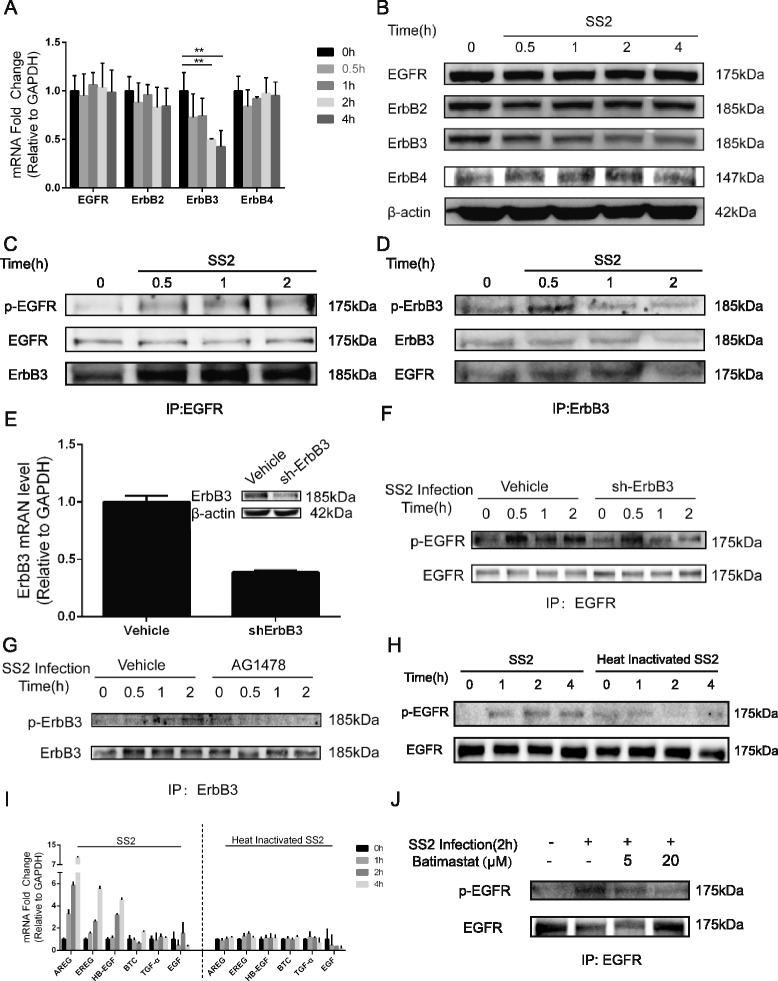
SS2 infection of hBMEC induces the ligand-dependent transactivation as well as the dimerization of EGFR. **a** Total RNAs extracted from hBMEC were analyzed by real-time PCR for the transcription of ErbB family genes. GAPDH was used as an internal control for normalization. **b** Immunoblot analysis of ErbB family members in whole cell extracts after infection of hBMEC with SC19 at an MOI of 10 for the indicated times. β-actin in cell lysates was analyzed as normalization control. **c**, **d** EGFR or ErbB3 was immunoprecipitated and immunoblotted with an anti-phosphotyrosine antibody (4G10) and then with the anti-EGFR or anti-ErbB3 antibody (*top* and *middle*). After stripping, the blots were reprobed with anti-ErbB3 or anti-EGFR antibodies to determine the possible interaction of EGFR with ErbB3. **e** Determination of ErbB3 expression in hBMEC transfected with either vehicle control or ErbB3 shRNA. **f** Knocking down of ErbB3 via shRNA attenuated SS2-induced EGFR activation. **g** AG1478 treatment significantly inhibited SS2-stimulated ErbB3 activation in hBMEC. **h** Heat-inactivated SC19 was unable to induce transactivation of EGFR, compared with the time-dependent activation of EGFR in response to viable SS2. **i** The induction of EGFR-related ligands in hBMEC in response to viable or heat-inactivated SS2 were compared by real-time PCR. GAPDH was used as the internal control. **j** Pretreatment with batimastat decreased SS2-induced transactivation of EGFR

Since AG1478 effectively inhibited the tyrosine phosphorylation of cellular proteins, we focused on the EGFR, a member of the receptor tyrosine kinase family. We first checked the transcription of these ErbB members by real-time PCR and found that the transcription levels of EGFR, ErbB2, and ErbB4 did not significantly change in response to SC19 infection, while ErbB3 showed a significant decrease since 2 h post infection (Fig. 3a). Similar results displaying a decrease of ErbB3 were obtained using western blotting (Fig. 3b). Subsequently, the phosphorylation of EGFR upon infection was investigated through immunoprecipitation with anti-EGFR antibody. The result showed a time-dependent phosphorylation of EGFR with SC19 infection (Fig. 3c). Notably, we observed a corresponding increase of ErbB3 in the EGFR immunoprecipitation samples, accompanied with the activation of EGFR (Fig. 3c). This prompted us to hypothesize that ErbB3 might be recruited by activated EGFR, thus forming EGFR/ErbB3 heterodimers. However, although ErbB2 is mostly reported to be an EGFR heterodimerization partner [37], we failed to detect its association, as well as that of ErbB4, with activated EGFR in response to SC19 infection. We similarly observed the inverse result after immunoprecipitation with an anti-ErbB3 antibody, showing that EGFR binding was increased upon the phosphorylation of ErbB3 (Fig. 3d), which further strengthened our hypothesis on the formation of EGFR/ErbB3 heterodimers in response to SS2 infection. Additionally, ErbB3 was knocked down in hBMEC via transfection with ErbB3 specific shRNA plasmid. As shown in Fig. 3e, both the transcription and the translation of ErbB3 decreased markedly in hBMEC. We further demonstrated that the time-dependent phosphorylation of EGFR in response to SC19 infection became attenuated in sh-ErbB3 transfected cells (Fig. 3f), and vice versa, 5 μM AG1478 treatment significantly blocked the infection-induced phosphorylation of ErbB3, compared with the vehicle control (Fig. 3g). This evidence confirmed our hypothesis that SS2 infection of hBMEC induced the formation of EGFR/ErbB3 heterodimers.

EGFR activation occurs via certain kinases and/or transactivation through binding to specific ligands. Several ligands are responsible for the activation of EGFR, including EGF, heparin-binding HB-EGF, amphiregulin (AREG), betacellulin (BTC), epiregulin (EREG), and transforming growth factor α (TGF-α). These EGFR-related ligands are proteolytically cleaved by the matrix metalloproteinase (MMP) family and bind to the extracellular ligand-binding domain to stimulate the dimerization of EGFR [38, 39]. To further investigate if SC19-induced EGFR activation is ligand-dependent, the EGFR phosphorylation in hBMEC incubated either with viable SC19 or with heat-inactivated SC19 were compared. As demonstrated, the heat-inactivated SC19 could not induce a time-dependent phosphorylation of EGFR as observed for viable SC19 cells (Fig. 3h). More importantly, the quantitative real-time PCR data supported that viable SC19 infection of hBMEC could stimulate the upregulation of AREG, EREG, and HB-EGF, while the heat-inactivated SC19 was unable to upregulate these ligands (Fig. 3i). Furthermore, when the MMP inhibitor Batimastat was used before SC19 infection, a dose-dependent attenuation of EGFR activation was observed (Fig. 3j). These suggest that SC19 infection of hBMEC could induce the transactivation of EGFR in a ligand-dependent manner.

### Inhibition of EGFR does not significantly affect bacterial adherence of hBMEC in vitro and bacterial colonization in vivo

**Fig. 4:**
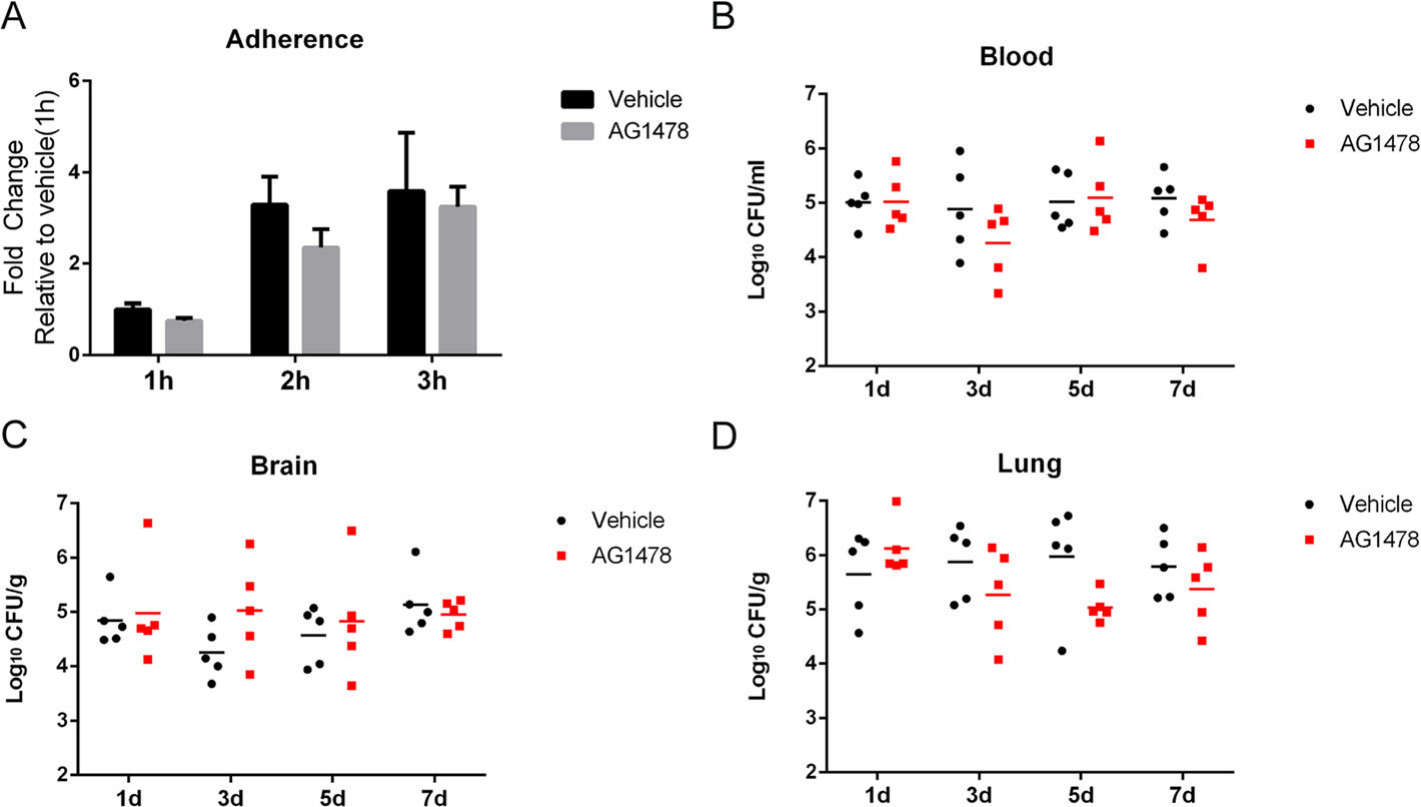
Effects of EGFR inhibition on SS2 adherence and colonization. **a** AG1478 pretreatment (5 μM) did not significantly affect SC19 adhesion to hBMEC in vitro. **b**–**d** Mice (*n* = 5) were administrated with AG1478, or DMSO as vehicle control, and then challenged with 2 × 10^8^ CFU SC19 strain. At the indicated time points, bacterial colonization in the blood, brain, and lung from both groups of mice were compared

To clarify the effects of EGFR in SS2-induced meningitis, we first investigated the adhesion of SS2 strain SC19 to hBMEC, with or without pretreatment of the EGFR inhibitor AG1478. There was no significant attenuation of SS2 adhesion after AG1478 treatment, compared with that of the vehicle control (Fig. 4a). In vivo, bacterial colonization in multiple tissues, such as the blood, brain, or lung, did not show any significant differences between the mice with and without AG1478 administration over a 7-day observation period (Fig. 4b–d). These together suggest that inhibition of EGFR does not affect SS2 strain adhesion of hBMEC, as well as bacterial penetration of the brain.

### SS2-induced strong neuroinflammation could be significantly prevented by AG1478

**Fig. 5:**
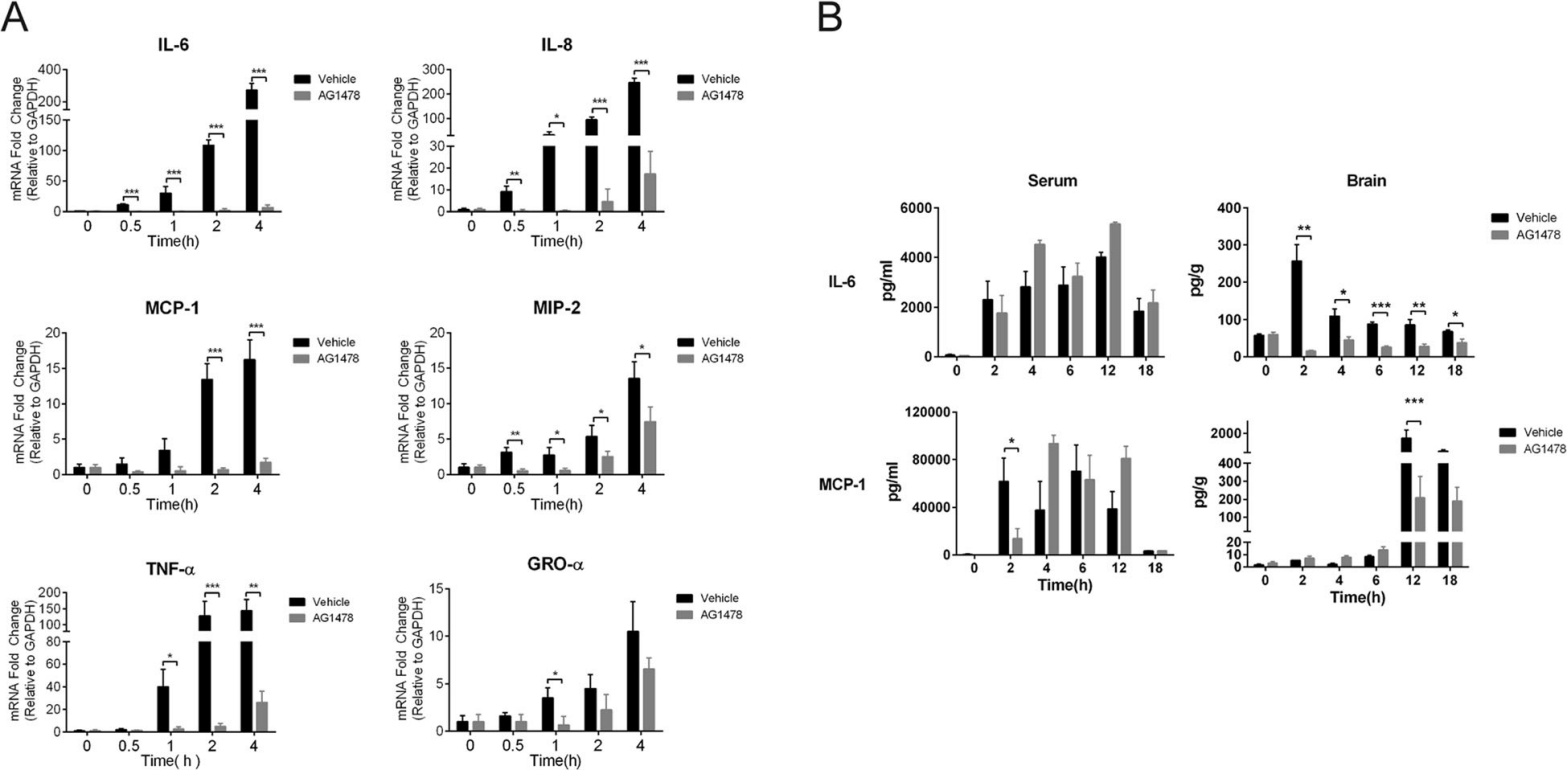
EGFR selective inhibitor AG1478 could significantly attenuate the SS2-induced neuroinflammation. **a** SC19 infection of hBMEC induced a time-dependent increase of the proinflammatory cytokines and chemokines, while this could be significantly blocked by the treatment of AG1478. **b** Serum and brains lysates were collected from challenged mice with or without AG1478 pretreatment. The levels of representative cytokine and chemokine, IL-6 and MCP-1, were determined and compared. Results were expressed as mean ± SD of three infected mice in each group

As previous studies have supported the association of SS2 infection with a strong inflammatory response in vivo [40, 41], we then investigated if EGFR participated in the neuroinflammatory process in response to SC19 infection. The hBMEC was pretreated with or without 5 μM AG1478 for 2 h prior to SC19 stimulation, and the quantitative real-time PCR data showed that bacteria-induced production of cytokines and chemokines (e.g., IL-6, IL-8, MCP-1, MIP-2, TNF-α, and GRO-α) were almost significantly inhibited by the treatment of AG1478 (Fig. 5a). Likewise in vivo, IL-6 and MCP-1 were selected as the representative cytokine and chemokine, and AG1478 significantly decreased the upregulation of IL-6 and MCP-1 in the brain (Fig. 5b, right panels). However noticeably, this inhibition did not effectively prevent the production of these cytokines and chemokines in the serum (Fig. 5b, left panels). These observations suggest that blocking EGFR activity with AG1478 could specifically and significantly alleviate the infection-induced inflammation in the central nervous system.

### SS2-induced EGFR transactivation contributes to neuroinflammation through MAPK-ERK1/2 and NF-κB signaling pathways

**Fig. 6:**
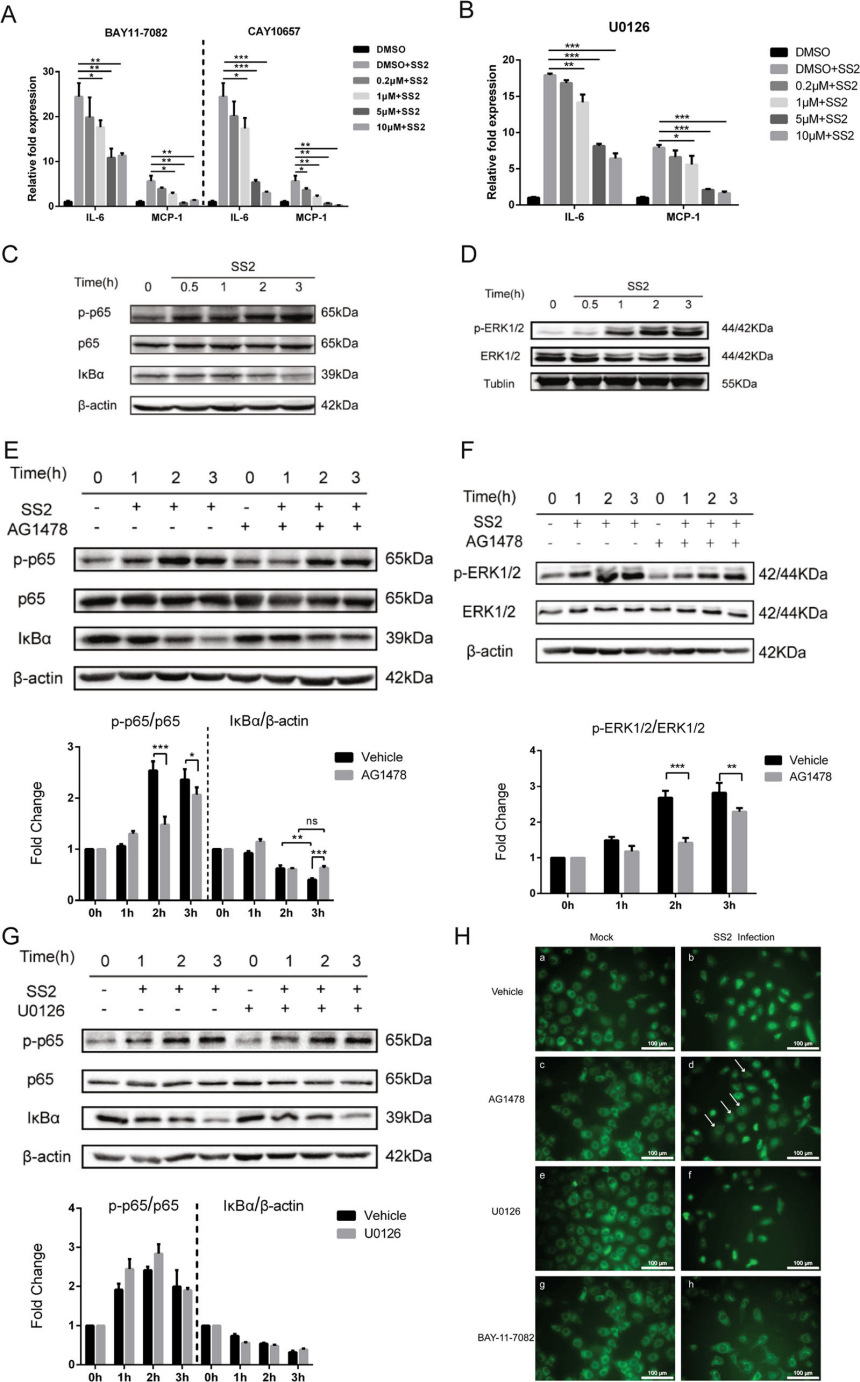
SS2-induced transactivation of EGFR contributes to the neuroinflammation via MAPK-ERK1/2 and NF-κB signaling pathways. **a**, **b** NF-κB inhibitors BAY11-7082 or CAY10657 and the ERK1/2 inhibitor U0126 dose-dependently inhibited SS2-induced upregulation of IL-6 and MCP-1. **c** SC19 infection of hBMEC induced an obvious phosphorylation of p65 subunit, as well as the degradation of IκBα. **d** SC19 infection of hBMEC induced obvious activation of ERK1/2. **e**, **f **EGFR-selective inhibitor AG1478 could significantly decrease the SC19-induced NF-κB signaling (including p65 phosphorylation and IκBα degradation), as well as ERK1/2 phosphorylation. **g** Blockage of ERK1/2 signaling by U0126 did not affect SS2-induced p65 phosphorylation and IκBα degradation. **h** Fluorescence microscopy of SS2-induced p65 subunit translocation to the nucleus in hBMEC with pretreatment of AG1478 (5 μM), U0126 (5 μM), or BAY-11-7082 (5 μM). Cells were incubated with anti-p65 antibody and visualized with a FITC-labeled goat anti-mouse IgG antibody. *Scale bar* = 100 μm

Above we have presumed the specific involvement of EGFR in the central inflammatory response. Next, we sought to determine the possible signaling pathway involving EGFR in the regulation of this inflammatory response. Previous research suggested that NF-κB signaling pathway plays an important role in the inflammatory response, and ERK1/2 was reported to be a common downstream signaling molecule of EGFR [9, 42]. We therefore used two NF-κB specific inhibitors BAY11-7082 and CAY10657, as well as the ERK1/2 inhibitor U0126, to analyze their effects on the generation of proinflammatory cytokines and chemokines. We found that both BAY11-7082 and CAY10657 could significantly, and dose-dependently, reduce the expression of IL-6 and MCP-1 induced by SS2 strain SC19. Similarly, the ERK1/2 specific inhibitor U0126 also decreased the upregulation of these two proinflammatory factors in a dose-dependent manner (Fig. 6a, b). Subsequently, we observed that the p65 subunit was phosphorylated time-dependently in response to SC19 infection, and correspondingly, IκBα was degraded with infection (Fig. 6c). Furthermore, ERK1/2 showed increasing phosphorylation in response to bacterial challenge (Fig. 6d). Together, these findings indicate the activation of NF-κB signaling and MAPK-ERK1/2 cascade in SS2 strain infection of hBMEC, and both NF-κB and MAPK-ERK1/2 signaling are involved in SS2 induction of proinflammatory factors. We further analyzed whether these two signaling events are affected by EGFR. The data shown in Fig. 6e, f demonstrated that blocking EGFR activity by pretreatment with AG1478 could significantly attenuate the activation of NF-κB p65 subunit and ERK1/2, as well as retarding the IκBα degradation by SS2 infection. Moreover, using immunofluorescence microscopy, we observed p65 translocation to the nucleus upon SS2 infection (Fig. 6h, panels a and b), and this nuclear translocation could be partly prevented by AG1478 pretreatment (Fig. 6h, panels c and d, white arrows indicated). Curiously, we wondered if MAPK-ERK1/2 signaling has some interaction with the p65 phosphorylation. After treatment with or without the ERK1/2 inhibitor U0126, we did not observe a marked difference in p65 phosphorylation, or IκBα degradation, upon SS2 infection (Fig. 6g). Moreover, the pretreatment with U0126 did not block the p65 translocation to the nucleus upon SS2 infection (Fig. 6h, panels e and f). Together, these findings indicate that NF-κB activation and the MAPK-ERK1/2 signaling cascade are two independent pathways that regulate the expression of proinflammatory cytokines and chemokines, although both are influenced by EGFR activity.

## Discussion

As an important zoonotic bacterial pathogen that causes great economic losses in animal husbandry, as well as public health problems, SS2 has received increasing attention and has been recognized as the third-largest contributor causing meningitis in adults [2, 3, 8, 43]. However, the mechanisms involved in SS2 strains penetration of the BBB and induction of CNS inflammation are not well understood. Studies have previously reported the important roles of several SS2 virulence factors in bacterial penetration of the BBB, for example, the capsular polysaccharides (CPS) regulate the invasion rate in meningeal cells and astrocytes, and encapsulated strains can induce exaggerated inflammatory responses [44]. The subtilisin-like protease SspA can induce IL-1β, IL-6, TNF-α, CXCL8, and CCL5 secretion dose-dependently in macrophages [12]. Enolase can significantly increase the production of IL-8 in the serum and brain and increase the permeability of the BBB [45]. In addition, suilysin was reported to contribute to cytoskeleton remodeling, and muramidase-released protein (MRP) could bind to human fibrinogen, therefore contributing to the breakdown of the BBB [4, 11]. Noticeably, these studies mainly focused on the virulence factors of SS2 strains and elucidated their possible functions in bacterial infection of the BBB. However, characterization of the host targets that are exploited by SS2 strains in the development of meningitis is less reported.

During SS2 induction of meningitis, bacterial binding of host extracellular matrix proteins of BMEC is the first step necessary for its successful infection [46]. SS2 association with BMEC can stimulate the activation of the vascular endothelia, which leads to a series of alterations and finally causes the CNS inflammatory response [47, 48]. Pathogen-induced CNS inflammation has been proven to be vital for the development of meningitis. For example, *pneumococcus* proliferation and immune recognition of bacterial components induced a strong inflammatory response, finally leading to BBB impairment [49, 50]. As a pathogen characterized by sepsis and meningitis, SS2 infection can induce the upregulation of diverse cytokines and chemokines, thus mediating the CNS inflammation storm which results in the alteration of the BBB permeability. The SS2 penetration of the disrupted BBB therefore accelerates the occurrence of meningitis [47, 51]. Here, a similar situation was observed with our SS2 strain SC19. The cytokine and chemokine concentration increased significantly in the blood after 2 h of infection, indicating an acute infection-induced inflammatory response against SC19. While the cytokines and chemokines maintained a high level in the blood for some time, they only began to be markedly increased several hours later in the brain, which contributes to the neuroinflammatory response. These findings imply that a certain level of systemic cytokines and chemokines in the blood is necessary for the induction of the meningitis and will determine the strength of the inflammation in the brain. Moreover, a previous study showed that intraperitoneal infection of SS2 could induce a rapid increase of systemic inflammatory factors in mice, including TNF-α, IL-6, IL-12, IFN-γ, IL-1β, CXCL1/KC/GRO-α, CCL2/MCP-1, and CCL5/RANTES, most of which reached a peak after 6 h of infection and subsequently decreased along with the infection [9]. Here, in vivo, we similarly demonstrated the rapid increase of proinflammatory cytokines and chemokines in the brain and serum, including IL-6, IL-1α, MCP-1, MIP-2, and GRO-α, suggesting the ability of SS2 to induce peripheral as well as CNS inflammation. Moreover, we picked IL-6 and MCP-1 as the representative cytokine and chemokine, respectively, and found that their induction in the brain could be significantly decreased by pretreatment with the EGFR inhibitor AG1478, although this treatment did not affect their induction in the serum by infection. Since IL-6 and MCP-1 were reported to be able to alter tight junction expression [52, 53], this finding implies that AG1478 might specifically protect the brain from cytokine/chemokine-mediated BBB disruption in early infection. Likewise, in vitro, we observed the high production of IL-6, IL-8, TNF-α, MCP-1, MIP-2, and GRO-α in hBMEC upon SS2 infection in a time-dependent manner, which similarly could be inhibited by AG1478. Notably, we observed a high level of MCP-1 in the serum as early as 2 h after SS2 infection, which was maintained until 12 h post infection. In contrast, the MCP-1 level in the brain upon SS2 infection was low for as long as 12 h post infection and then exhibited a sudden increase. MCP-1 is vital for monocyte recruitment and amplification of inflammation, and previous studies demonstrated decreased BBB leakage, as well as astrogliosis and macrophage/microglia accumulation in MCP-1 KO mice [54, 55]. Whether MCP-1 could be a promising biomarker indicating SS2 meningitis should be further investigated. Thus, altogether, our observations supported that EGFR plays an important role in SS2 strain SC19-induced meningitis, in which it can influence the SS2 induction of the cytokines and chemokines in the brain.

In our work, we demonstrated that SS2 activated EGFR by increasing the shedding of the EGFR-associated ligands AREG, EREG, and HB-EGF, rather than interacting directly with EGFR. Previous studies show that AREG can upregulate mucin gene expression in obstructive airway diseases, EREG modulates Toll-like receptor (TLR)-mediated immune responses, and HB-EGF overexpression promotes vascular endothelial growth factor (VEGF) signaling resulting in hydrocephalus [56–58]. These ligands could induce the rapid phosphorylation of EGFR and subsequent intracellular signal transduction. Additionally, different ligands induce different dimerization of ErbBs, thus mediating different signal transduction and outcomes [59]. Human cytomegalovirus induces the heterodimerization of EGFR-ErbB3, which was proven to be required for virus invasion [17]. *Neisseria meningitidis* activation of ErbB2 stimulates downstream signaling which affects cortical actin polymerization [19]. Here, we demonstrated the important role of EGFR and the formation of EGFR-ErbB3 heterodimers in hBMEC in response to SS2 challenge. Nevertheless, we could not exclude the possibility of EGFR homodimerization in response to SS2 infection based on our current data. Although ErbB2 is the preferred heterodimerization partner [37], we did not observe its activation, or association with EGFR, upon SS2 challenge. Therefore, we put forward the hypothesis that SS2 infection of hBMEC induces a ligand-dependent transactivation of EGFR and its dimerization with ErbB3, which leads to the activation of their intrinsic tyrosine kinase activity and regulates the production of proinflammatory cytokines and chemokines in the brain.

Bacterial pathogens exploit host cell signaling molecules to promote their infections, but the underlying mechanisms vary depending on specific pathogens and different host cells. As an important clinical therapeutic target and the signaling receptor on the cell surface, EGFR has been investigated in multiple bacterial infections. Transactivation of EGFR in *N. gonorrhoeae* infection can weaken the apical junction and polarity of epithelial cells, and thus enhance pathogen invasion [22, 60]. However, our results could not support a close relationship between EGFR activation and bacterial colonization in vivo and EGFR blockage could not attenuate the adherence of SS2 strain SC19 to hBMEC. In the field of inflammatory responses involving EGFR, *K. pneumoniae* could subvert host inflammation via the regulation of NF-κB signaling through an EGFR-dependent PI3K–AKT–PAK4–ERK–GSK3β pathway [23]. The nontypeable *H. influenza* induces activation of EGFR, which acts as a negative regulator of TLR2 expression via a Src-dependent p38 signaling pathway [24]. Here, we further highlight the involvement of EGFR in SS2-induced meningitis, by the demonstration that SS2-induced transactivation of EGFR promotes the MAPK-ERK1/2 as well as NF-κB signaling pathways in hBMEC, which subsequently initiates and mediates the inflammatory response in the brain and finally the CNS dysfunction.

## Conclusions

In this study, we provided evidence for the first time that SS2 infection of hBMEC could induce the ligand-dependent transactivation of EGFR that involves AREG, EREG, and HB-EGF, and the heterodimerization of EGFR with ErbB3. This EGFR transactivation triggers the downstream MAPK-ERK1/2 pathway, as well as NF-κB signaling, both of which mediate the production of proinflammatory cytokines and chemokines in the host brain, thus contributing to neuroinflammation and the development of SS2 meningitis. These findings support the important role of EGFR and its working mechanism in SS2-induced meningitis and suggest a potential regulatory mechanism in which EGFR may represent a promising therapeutic target that mediating the CNS immune response.

## Abbreviations

(IFN)-γ: Interferon-gamma
AREG: Amphiregulin
CCL5/RANTES: C-C motif chemokine ligand 5
CNS: Central nervous system
EGFR: Epidermal growth factor receptor
EREG: Epiregulin
ERK: Extracellular regulated protein kinases
GAPDH: Glyceraldehyde-3-phosphate dehydrogenase
GRO-α/CXCL1: Chemokine (C-X-C motif) ligand 1
HB-EGF: EGF-like ligand
hBMEC: Human brain microvascular endothelial cell
IL: Interleukin
MCP-1: Monocyte chemoattractant protein-1
NF-κB: Nuclear factor kappa B
SS2: *Streptococcus suis* serotype 2
TNF-α: Tumor necrosis factor-alpha
